# Clinical Trials for Gene Therapy in Lysosomal Diseases With CNS Involvement

**DOI:** 10.3389/fmolb.2021.624988

**Published:** 2021-09-16

**Authors:** Caroline Sevin, Kumaran Deiva

**Affiliations:** Pediatric Neurology Department, Hôpital Bicêtre, Le Kremlin Bicêtre, France

**Keywords:** lysosomal diseases, gene therapy, Adeno-associated virus, lentival vector, CNS-central nervous system

## Abstract

There are over 70 known lysosomal storage disorders (LSDs), most caused by mutations in genes encoding lysosomal hydrolases. Central nervous system involvement is a hallmark of the majority of LSDs and, if present, generally determines the prognosis of the disease. Nonetheless, brain disease is currently poorly targeted by available therapies, including systemic enzyme replacement therapy, mostly (but not only) due to the presence of the blood–brain barrier that restricts the access of orally or parenterally administered large molecules into the brain. Thus, one of the greatest and most exciting challenges over coming years will be to succeed in developing effective therapies for the treatment of central nervous system manifestations in LSDs. Over recent years, gene therapy (GT) has emerged as a promising therapeutic strategy for a variety of inherited neurodegenerative diseases. In LSDs, the ability of genetically corrected cells to cross-correct adjacent lysosomal enzyme-deficient cells in the brain after gene transfer might enhance the diffusion of the recombinant enzyme, making this group of diseases a strong candidate for such an approach. Both *in vivo* (using the administration of recombinant adeno-associated viral vectors) and *ex vivo* (auto-transplantation of lentiviral vector-modified hematopoietic stem cells-HSCs) strategies are feasible. Promising results have been obtained in an ever-increasing number of preclinical studies in rodents and large animal models of LSDs, and these give great hope of GT successfully correcting neurological defects, once translated to clinical practice. We are now at the stage of treating patients, and various clinical trials are underway, to assess the safety and efficacy of *in vivo* and *ex vivo* GT in several neuropathic LSDs. In this review, we summarize different approaches being developed and review the current clinical trials related to neuropathic LSDs, their results (if any), and their limitations. We will also discuss the pitfalls and the remaining challenges.

## Introduction

Lysosomal storage disorders (LSDs) are caused by mutations in genes encoding hydrolases or proteins involved in lysosomal transport, biogenesis, or maturation (Platt et al., 2018; Marques and Saftig, 2019; Martina et al., 2020), resulting in significant perturbations of lysosomal homeostasis. Individually, LSDs are classified as rare diseases, but overall, they affect one in 5,000–7,000 live births worldwide and represent a major health problem. Moreover, the true prevalence is likely higher, due to misdiagnosed/undiagnosed cases (Giugliani et al., 2017). LSDs are mostly autosomal recessive, rarely X-linked. They were historically considered “storage disorders,” due to the deficiency of lysosomal hydrolases with resulting impaired degradation and accumulation of complex substrates, and classified according to the nature of the material stored (mucopolysaccharidoses, sphingolipidoses, glycoproteinoses, glycogen storages diseases, or lipid storage diseases). Nonetheless, due to the emergence of diseases involving other lysosomal functions and recent understanding of their molecular basis, a more recent and complete classification has emerged (Boustany, 2013). In addition to the defect in lysosomal hydrolases, it covers nonenzymatic lysosomal protein defects; transmembrane protein defects (transporters and structural proteins); lysosomal enzyme protection, posttranslational processing, or trafficking defects; polypeptide degradation defects; and neuronal ceroid lipofuscinoses (NCLs) (Filocamo and Morrone, 2011; Huizing and Gahl, 2020). According to the WORLDSymposia^®^ official list of lysosomal diseases, 68 clinical conditions related to 57 distinct genes are recognized as LSDs, including 50 enzyme deficiencies (https://worldsymposia.org/official-list-of-lysosomal-diseases/).

LSDs, in addition to their multiple origins, are multisystemic diseases and can lead to dysfunction in various peripheral tissues and organs, with a broad spectrum of clinical manifestations determined by the degree of protein dysfunction, the biochemical nature and distribution of the material stored in organs and cell types, cell turnover/metabolism, and other genetic or epigenetic factors [for review, see (Boustany, 2013; Ferreira and Gahl, 2017; Platt et al., 2018; Sun, 2018)]. Clinical features include, but are not limited to, neurologic symptoms, organomegaly, coarse facial features, bone abnormalities (dysostosis multiplex), cardiac involvement, visual and hearing deficits, and ocular signs (corneal clouding or macular cherry-red spots). The diagnosis of LSDs is based on the combination of clinical symptoms, paraclinical examinations [fundus, brain magnetic resonance imaging (MRI), bone x-rays, abdominal and cardiac ultrasound, electrophysiology, tissue biopsy, *etc*.], detection of the stored product (mostly in urine), enzyme activity or protein expression, and molecular studies (screening for mutation of a specific gene, gene panel, and whole exome sequencing) (Wenger et al., 2003; Poswar et al., 2019; Fuller, 2020).

Though LSDs typically emerge in infancy and childhood, the age at first symptoms and spectrum of clinical signs vary from the neonatal period to adulthood, with a continuum of disease severity (Platt et al., 2018; Marques and Saftig, 2019). Patients are generally classified based on the age of clinical onset, as congenital or infantile (which usually have the most severe presentation), late infantile, juvenile, and adult forms (most often milder phenotypes). While disease severity does not fully correlate with genotype and residual activity, the degree of residual function of the defective protein usually influences the age of symptom onset. In particular, patients null for a given protein present symptoms at birth or in early infancy, whereas milder mutations lead to juvenile or adult-onset forms.

More than two-thirds of LSDs share neurological involvement which is often at the forefront and leads to progressive and often severe neurodegeneration. Central nervous system (CNS) involvement tends to determine the prognosis of the disease and remains the most important unmet therapeutic challenge, it being the main cause of morbidity and early mortality (Giugliani et al., 2018; Pará et al., 2020). CNS disease manifests as intellectual disability or regression of developmental milestones, seizures, ataxia, pyramidal/extrapyramidal signs, behavioral abnormalities, dementia, and other neurological defects (Jardim et al., 2010; Maegawa, 2019). Neurodegeneration may occur in various CNS areas with temporospatial changes and differential CNS cell susceptibility to storage (Platt et al., 2012). Both gray and white matter may be involved (Pará et al., 2020).

Pathophysiology of lysosomal diseases is complex and only partially deciphered. An abundance of generated mouse models and naturally occurring large animal models for LSDs have proven to be crucial for understanding the pathogenesis of these disorders and evaluating new treatments (Pastores et al., 2013; Gurda and Vite, 2019). More recently, the development of induced pluripotent stem cells (iPSCs) has provided additional important tools directly derived from human materials (Zunke and Mazzulli, 2019; Kido et al., 2020). Nonetheless, the pathophysiology of LSDs remains incompletely understood.

Despite LSDs differing from each other in several disease-specific features, they share some similar pathogenic mechanisms. Lysosomes are involved in macromolecule breakdown/recycling and signaling, and are a key for nutrient sensing, ion homeostasis, and calcium signaling (Platt et al., 2012; Platt et al., 2018). They fuse with endosomes, phagosomes, and plasma membrane and are in a continuous cross talk with other organelles (mitochondria, peroxisomes, nucleus, *etc*.) through contact sites (Platt et al., 2018). Consequently, an accumulation of undegraded substrates or impaired transport of molecules leads to lysosomal dysfunction and cellular damage.

If we focus on the CNS, the first event in most LSDs is the accumulation of specific undegraded compounds inside organelles of the endosomal–autophagic–lysosomal system. Secondary storage of different substrates may occur, due to the secondary inhibition of other lysosomal proteins. The accumulation of primary and secondary substrates triggers a cascade of damaging events that may impact lysosomes and other organelles, leading to an overall cell dysfunction which impairs membrane repair, autophagy, exocytosis, lipid homeostasis, signaling cascades, and finally cell viability (Marques and Saftig, 2019; Schultz et al., 2011; Ballabio and Gieselmann, 2009). Impaired mitochondrial function, oxidative stress, perturbation of calcium homeostasis, microglia activation, and astrogliosis contribute greatly to the pathophysiology of LSDs and associated neurodegeneration (Platt et al., 2012; Vitner et al., 2010; Plotegher and Duchen, 2017; Ballabio and Bonifacino, 2020). Among pathophysiological factors, neuroinflammation is a hallmark of many LSDs with CNS involvement and has emerged as a key factor in promoting neurodegeneration in these diseases, as in other neurodegenerative diseases. Although the strength of inflammatory changes and the mechanisms responsible for triggering neuroinflammation are different for each disease, microglial activation and astrogliosis generate a neurotoxic environment through the secretion of cytokines, chemokines, and proapoptotic molecules that may negatively impact CNS cell survival and contribute to the brain pathology (Bosch and Kielian, 2015; Rigante et al., 2017). Importantly, it has been shown that microglial activation may occur even before the appearance of typical pathological signs (Wada et al., 2000; Fiorenza et al., 2018). Additionally, leakage of the blood–brain barrier (BBB) allows the infiltration of peripheral immune cells that may amplify/modify intrinsic immune reactions in the CNS (Grabowski, 2017; Rigante et al., 2017). Thus, beyond the replacement of a missing enzyme or a defective gene, the correction of neuroinflammation, as well as other secondary pathological mechanisms, will likely be crucial for any therapeutic option to be fully successful in neuropathic LSDs.

Overall, LSDs are progressive and complex, with a variable rate of progression and severity. There is often a “window of opportunity” for therapeutic intervention, in which patients are presymptomatic or have mild symptoms, likely being more amenable to any treatment. Consequently, early diagnosis and intervention before the onset of irreversible neuropathology may provide substantial benefits, whereas supportive care is most often the only available option for patients with advanced disease, particularly for the affected CNS. This emphasizes the need for a broad familial screening once an index case is diagnosed in a family, to detect the disease in pre-or early-symptomatic siblings.

### Current Treatment Options for Central Nervous System Disease in Lysosomal Storage Disorders

Apart from the CNS involvement, some LSDs are treatable, at least partially. The missing or defective enzyme can be provided exogenously through intravenous enzyme replacement therapy (ERT), the amount of stored material can be reduced by substrate reduction therapy (SRT), chaperone molecules can improve the function of the defective enzyme, and hematopoietic stem cell transplantation (HSCT) may modify the natural history of some LSDs (Brady, 2006; Beck, 2018; Platt, 2018; Poswar et al., 2019; Thomas and Kermode, 2019; Lachmann, 2020).

Both ERT and allogeneic HSCT, which remain the standard of care for LSDs, take advantage of the secretion and receptor-mediated endocytosis capacity of lysosomal enzymes. Physiologically, a small percentage (approximately 10%) of lysosomal enzymes escape the endosomal/lysosomal compartment and are secreted in the extracellular space where they may be taken up by adjacent cells *via* mannose-6-phosphate receptor-dependent or -independent pathways (Sands and Davidson, 2006; Coutinho et al., 2012; Staudt et al., 2017). Thus, after intravenous ERT administration, the uptake of recombinant enzyme is fundamentally from the bloodstream by cells and addressed to their lysosome. In HSCT or gene therapy (GT) approaches, deficient cells will be corrected through the phenomenon of *cross-correction*, that is, the ability of lysosomal enzyme-expressing cells to correct others that are enzyme deficient. As it is traditionally claimed that increasing the level of enzyme activity in cells to 15–20% of control values could be enough to correct the disease, a relatively small number of cells expressing (through HSCT) or over-expressing (through GT) the missing enzyme may be sufficient to correct numerous deficient cells in the brain (Sands and Davidson, 2006) and be associated with a significant impact on the clinical course of the disease. Importantly, however, the cross-correction efficacy will depend on the “power of secretion” of the lysosomal enzyme by the cell type in which it is produced, and on its capacity to be recaptured by targeted cellular types.

Intravenous ERT is inefficient for targeting the CNS component of the disease because the circulating enzyme does not cross the BBB. Various strategies have been developed to overcome this problem and allow delivery of therapeutic agents to the brain, including opening the BBB by ultrasound, transforming the therapeutic enzyme into molecules capable of crossing the BBB, and administering the enzyme directly into the cerebrospinal fluid (CSF) (Bellettato and Scarpa, 2018). Intra-CSF administration (intrathecal or intracerebroventricular) has been evaluated in clinical trials for several LSDs [MPSIIIA (Wijburg et al., 2019), MPSI (Eisengart et al., 2019), MPSII (Muenzer et al., 2016), or MLD (í Dali et al., 2020)], suggesting some degree of effect, but clearly insufficient to prevent/stabilize the disease. On the other hand, encouraging results were obtained after the intracerebroventricular administration of recombinant enzyme in patients with CLN2 disease, if treated at an early stage of their disease (Schulz et al., 2018).

HSCT may notably modify the natural history and improve CNS-related deficits in some LSDs, if delivered early enough. The mechanism of action is based on the fact that, following HSC transplantation, a fraction of donor-derived cells from the myelomonocytic lineage are able to migrate to the CNS, and then locally differentiate to form perivascular and parenchymal (resting) microglia (Asheuer et al., 2004). Microglial cells are continuously replenished by bone marrow-derived myeloid precursors present throughout the entire CNS (Prinz et al., 2019). Donor cells can both mediate the cross-correction of the lysosomal enzyme in neighboring neuronal and glial cells and/or act directly on the neuroinflammatory process. HSCT is recommended for patients with Hurler disease (MPSI) if they are younger than 2·5 years, in combination with intravenous ERT (De Ru et al., 2011; Parini et al., 2017). It is also proposed for patients with metachromatic leukodystrophy (MLD) and Krabbe disease, if performed presymptomatically in early-onset forms (Wright et al., 2017) or in pre-or early-symptomatic patients in late-onset forms of the disease (Boucher et al., 2015; Groeschel et al., 2016). If patients are at an advanced stage of their disease, this treatment is unsatisfactory, likely due in part to an insufficient level of production of the enzyme by donor cells in the CNS, in addition to a delayed action (over 9–12 months) linked to a slow turnover of microglia. Another treatment option is the use of HSCs genetically modified using lentiviral GT vectors to overexpress the therapeutic enzyme (*ex vivo* GT, see below).

In summary, intrathecal ERT could be helpful and act very quickly, but such treatment would require administration of a sustained level of recombinant protein and lifelong repeated injections with some risk of infections related to the need for an intrathecal or intraventricular device to target CNS pathology. HSCT has demonstrated positive effects in presymptomatic or early-symptomatic patients only in a few numbers of LSDs, and its effect is hampered by a delayed onset of action, especially for rapidly progressing diseases and/or for already symptomatic patients. Thus, even if both therapeutics are powerful tools to modify the natural history of CNS disease in some LSDs, they may be insufficient to arrest or correct the neuropathology, once already present. Combining both treatments is certainly an interesting approach for those LSDs in which HSCT has shown some degree of therapeutic effect. Finally, it should be mentioned that these therapies are limited to LSDs involving secreted lysosomal enzyme. Based on these comments, additional therapeutic strategies—including gene therapy—should clearly be assessed to increase the therapeutic arsenal of these LSDs with neurological impairment.

### Gene Therapy for Lysosomal Storage Disorders With Neurologic Involvement

Correction/prevention of brain disease would at least require substantial, constant, and long-lasting expression of the missing enzyme or protein in the right cells of the CNS (i.e., neurons, glial cells, or microglia, depending on the disease). Among promising therapeutic options, GT is attracting growing interest for the treatment of neurodegenerative diseases including LSDs, due to the availability of viral vectors able to target CNS cells and even cross the BBB, new methods for large-scale production and improvements in the technical conditions for administration (Piguet et al., 2017; Ingusci et al., 2019; Penati et al., 2017). The goal of GT is to achieve, after a “one-shot” procedure, long-lasting expression of the therapeutic gene, sufficient to improve or halt disease symptoms with minimal adverse events (High and Roncarolo, 2019). The principle of gene therapy in LSDs being based on the cross-correction capacity of transduced cells, this approach is mainly intended to treat lysosomal diseases involving enzymes, capable of being secreted by cells having integrated the vector and endocytosed by untransduced adjacent cells. There is an extensive literature providing evidence of its feasibility and efficacy in animal models of LSDs [for review see (Xu et al., 2016; Nagree et al., 2019; Favret et al., 2020; Liu et al., 2020)]. Indeed, various approaches are feasible, depending on the type of therapy (*ex vivo* or *in vivo*) and route of administration (peripheral or central). Adeno-associated viruses (AAVs) and lentiviral vectors (LVs) have emerged as the vectors of choice for GT of neurodegenerative diseases including LSDs (Piguet et al., 2017). For more details about the characteristics of these vectors, the reader may refer to the article by Edelman and Maegawa, which is part of this research topic on “Neurodegenerative Lysosomal Storage Diseases (LSDs): Between Biology and Innovative Therapeutic Approaches”.

A search on PubMed using the keywords “gene therapy” and “neurodegenerative diseases” found more than 13,000 publications since 1990 and more than 8,000 over the last 10 years. If restricted to “lysosomal storage diseases” and “central nervous system”, more than 770 publications were found, nearly 250 over the last 5 years. There are currently around 40 clinical trials registered on clinicaltrial.gov (https://clinicaltrials.gov) using GT in LSDs, including long-term follow-up studies. These results demonstrate the growing interest in LSDs, their pathophysiological mechanisms (which share features with other neurodegenerative diseases) and their treatment, including GT. In this review, we will not discuss GT trials involving LSDs without major CNS involvement (such as Fabry disease, Gaucher disease type 1, Pompe disease, MPSVI, and cystinosis), for which the interventions available are intravenous administration of AAV vectors or HSCT-GT with LVs. Rather, we focus on clinical trials designed to target CNS diseases in LSDs, their results (if any), and their limitations. Encouraging results in preclinical studies obtained in rodents and large animal models have warranted the development of clinical trials in several LSDs with CNS involvement, namely, MLD, mucopolysaccharidosis (MPSIIIA, MPSIIIB, MPS II, and MPSI), GM1 gangliosidosis, and ceroid lipofuscinosis (CLN2, CLN3, and CLN6). For some of these, results have already been published, while others are still underway. Both *in vivo* and *ex vivo* approaches have been evaluated in LSDs (see Table 1).

**TABLE 1:** Completed or ongoing viral vector gene therapy-based clinical trials for lysosomal storage disorders with CNS involvement. Data from clinicaltrials.gov as of June 2021.

**NCT Number**	**Status**	**Conditions**	**Vector**	**Administration**	**Phases**	**Nb of patients**	**Completion Date**
**NCT04273269**	Not yet recruiting	GM1 gangliosidosis	AAVrh10	Intracisternal	Phase 1|phase 2	16	June 2025
**NCT03952637**	Recruiting	GM1 gangliosidosis	AAV9	Intravenous	Phase 1|phase 2	45	April 2024
**NCT04713475**	Not yet recruiting	GM1 gangliosidosis	AAV Hu68	Intracisternal	Phase 1|phase 2	20	February 2029
**NCT04693598**	Recruiting	Krabbe disease	AAVrh10 (combined with allo-HSCT)	Intravenous	Phase 1|phase 2	6	April 2023
**NCT04771416**	Not yet recruiting	Krabbe disease	AAV Hu68	Intracisternal	Phase 1|phase 2	24	January 2030
**NCT01560182**	Active, not recruiting	Metachromatic leulodystrophy (LI/EJ)	Lentiviral HSC-GT	*Ex vivo*	Phase 1|phase 2	20	April 2023
**NCT04283227**	Recruiting	Metachromatic leulodystrophy (LJ)	Lentiviral HSC-GT	*Ex vivo*	Phase 3	6	January 2032
**NCT03392987**	Active, not recruiting	Metachromatic leulodystrophy (LI/EJ)	Lentiviral HSC-GT	*Ex vivo*	Phase 2	10	August 2028
**NCT03725670**	Recruiting	Metachromatic leukodystrophy	Lentiviral HSC-GT	*Ex vivo*		10	November 2020
**NCT02559830**	Recruiting	Metachromatic leukodystrophy	Lentiviral HSC-GT	*Ex vivo*	Phase 1|phase 2	50	October 2025
**NCT01801709**	Active, not recruiting	Metachromatic leulodystrophy (LI/EJ)	AAVrh10	Intracerebral	Phase 1|phase 2	5	April 2019
**NCT03580083**	Recruiting	MPS I	AAV9	Intracisternal	Phase 1|phase 2	5	July 2023
**NCT03488394**	Active, not recruiting	MPS I	Lentiviral HSC-GT	*Ex vivo*	Phase 1|phase 2	8	January 2023
**NCT02702115**	Active, not recruiting	MPS I	AAV- ZFN-mediated genome editing delivery	Intravenous	Phase 1|phase 2	3	January 2022
**NCT03566043**	Recruiting	MPS II	AAV9	Intracisternal	Phase 1|phase 2	12	December 2023
**NCT04571970**	Recruiting	MPS II	AAV9	Intracisternal	Phase 1|phase 2	6	June 2023
**NCT00004454**	Completed	MPS II	T-lymphocytes, transduced with retrovirus L2SN	*Ex vivo*	Phase 1|phase 2	2	
**NCT03041324**	Active, not recruiting	MPS II	AAV- ZFN-mediated genome editing delivery	Intravenous	Phase 1|phase 2	9	February 2022
**NCT03300453**	Completed	MPS IIIB	AAV5	Intracerebral	Phase 1|phase 2	4	November 2019
**NCT03315182**	Recruiting	MPS IIIB	AAV9	Intravenous	Phase 1|phase 2	15	October 2022
**NCT03612869**	Active, not recruiting	MPS IIIA	AAVrh10	Intracerebral	Phase 2|phase 3	20	March 2022
**NCT04201405**	Recruiting	MPS IIIA	Lentiviral HSC-GT	*Ex vivo*	Phase 1|phase 2	5	October 2024
**NCT04088734**	Recruiting	MPS IIIA	AAV9	Intravenous	Phase 1|phase 2	12	December 2023
**NCT02716246**	Recruiting	MPS IIIA	AAV9	Intravenous	Phase 1|phase 2	22	December 2022
**NCT01474343**	Completed	MPS IIIA	AAVrh10	Intracerebral	Phase 1|phase 2	4	May 2013
**NCT02053064**	Completed	MPS IIIA	AAVrh10	Intracerebral	Phase 1|phase 2	4	June 2017
**NCT04669535**	Recruiting	Tay-sachs/Sandhoff	AAV9	IC + icis + IT	Phase 1	18	June 2028
**NCT04798235**	Recruiting	Tay-sachs/Sandhoff	AAV9	Intrathecal	Phase 1|phase 2	6	March 2027
**NCT01161576**	Completed	LINCL (CLN2)	AAVrh10	Intracerebral	Phase 1	12	December 2020
**NCT01414985**	Completed	LINCL (CLN2)	AAVrh10	Intracerebral	Phase 1|phase 2	8	February 2017
**NCT02725580**	Active, not recruiting	LINCL (CLN6)	AAV9	Intrathecal	Phase 1|phase 2	13	November 2021
**NCT03770572**	Active, not recruiting	NCL (CLN3)	AAV9	Intrathecal	Phase 1|phase 2	7	September 2023
**NCT04737460**	Recruiting	NCL (CLN7)	AAV9	Intrathecal	Phase 1	4	Februay 2029

#### *In vivo* Gene Therapy for Lysosomal Storage Disorders With Neurologic Involvement

For CNS-*in vivo* GT (i.e., direct introduction of the therapeutic vector into the CNS), AAVs have emerged as the safest vector and are the most commonly used (Kantor et al., 2014), allowing various routes of administration, with specific targeting capacities depending on the capsid choices, stable transgene expression in postmitotic cells, low immunogenicity and low risk of toxicity or insertional mutagenesis. The vector can be delivered directly into the brain (intracerebral) or into the CSF (intrathecal, intracerebroventricular, or intracisternal) (Bey et al., 2020). More recently, new serotypes of AAV have been shown to target the CNS after intravenous administration (in this case the vector has to cross the BBB), AAV9 and AAVrh10 being the most frequently used serotypes in clinical practice, due to their high capacity to transduce neural cells (Cearley et al., 2008; Piguet et al., 2017; Bey et al., 2020) and some ability to cross the BBB. More recently, new AAV serotypes have been engineered with high tropism for the CNS after intravenous administration (Li and Samulski, 2020). In addition to cross-correction, axonal transport of the enzyme through projections from transduced neurons may allow distribution of the enzyme across large areas of the brain (Passini et al., 2002; Luca et al., 2005).

Initial CNS GT trials in LSDs used intraparenchymal administration to bypass the BBB and deliver genes directly into the brain region of interest, with the goal of obtaining robust and diffuse transduction of brain cells (mainly neurons) associated with widespread cross-correction of non-transduced cells. Therapeutic vectors were delivered locally, to specific parenchymal brain regions, through neurosurgical stereotactic injections. Advantages of this route of administration include minimal biodistribution to peripheral organs (which may reduce immunogenicity) and substantially lower vector doses than those required for other routes of administration. In the early 2000 s, AAV2 was the first serotype used in Canavan disease and late infantile neuronal ceroid lipofuscinosis (LINCL), establishing the safety of the procedure, and some trends suggestive of a clinical effect (Worgall et al., 2008; Leone et al., 2012). The emergence of new serotypes (AAV5, AAV9, and AAVrh10), demonstrating better transduction and diffusion in the CNS, prompted researchers to pursue this approach and undertake clinical trials using these new vectors. The route of administration was either intraparenchymal, using AAVrh10 or AAV5 vectors (MLD, MPSIIIA, MPSIIIB, and Batten disease), intracisternal using AAV9 or AAVrh10 vectors (MPSI, MPSII, and GM1 gangliosidosis), or intravenous using AAV9 (MPSIIIA, MPSIIIB, and GM1 gangliosidosis).

### Intracerebral Gene Therapy for Late Infantile Neuronal Ceroid Lipofuscinosis

The AAVrh10 vector showed superiority over AAV2 in LINCL mice, as well as long-term expression and safety in rats and nonhuman primates (Sondhi et al., 2007; Sondhi et al., 2012). A clinical trial, seeking to assess the safety and efficacy of AAVrh.10 for delivering the *CLN2* gene to children in the early stages of the disease, is currently in progress (NCT01414985; NCT01161576). Primary endpoints are change in CNS function (measured by the Weill Cornell LINCL Scale) and safety (measured by MRI) in treated children, compared to a natural history cohort. The first results of this clinical trial have recently been published for eight children after 18 months follow-up. Results showed an 1.3-2.6-fold increase in cerebral spinal fluid TPP1 and a decrease in the progression of the disease (slower loss of gray matter volume in 4/7 children, significant reduction in the rate of decline of motor and language function). The authors concluded that the treatment lowered the progression of disease, but those improvements in vector design and delivery strategies will be needed to halt disease progression using gene therapy (Sondhi et al., 2020).

### Intracerebral Gene Therapy for Metachromatic Leukodystrophy

Metachromatic leukodystrophy (MLD, OMIM #250100) is a rare, autosomal recessive disease caused by deficient activity of the lysosomal enzyme arylsulfatase A (ARSA), resulting in sulfatide accumulation and subsequent demyelination and neuronal loss within the central and peripheral nervous systems (CNS and PNS). Three clinical forms of MLD have been described, based on the age of symptom onset (late infantile, juvenile, and adult forms) (Gieselmann and Krägeloh-Mann, 2010; Van Rappard et al., 2015). In the case of late-onset forms (first symptoms after 4 years), which are more variable and progress more slowly, HSCT may modify their natural history if performed in presymptomatic or early-symptomatic patients (Groeschel et al., 2016). In the early-onset forms (first symptoms before 4 years), the disease progresses very rapidly towards severe motor and cognitive regression and premature death, no available therapy being effective once patients are symptomatic. This prompted us to develop an *in vivo* GT strategy in children with early-onset MLD, using an AAVrh10-ARSA vector. Preclinical results have shown the feasibility and efficacy of this approach and allowed us to move toward its use in clinical practice (Piguet et al., 2012; Zerah et al., 2015). In a phase I–II clinical trial, four MLD children (aged between 9 months and 5 years, and either presymptomatic or early-symptomatic) received stereotactic intracerebral injections of an AAVrh10-ARSA vector at 12 sites in the white matter of the centrum semiovale (10E12 to 4x10E12 vector genome). Patients were not immunosuppressed. The neurosurgical procedure and treatment were well tolerated. There was no evidence of a cellular immune response against the ARSA transgene, though localized hyperintense T2-weighted signals were detected around the injection sites from M+3 postinjection in some patients that remained stable thereafter without clinical impact. Despite the long-lasting restoration of ARSA activity in the CSF (to 20–70% of values in controls), treatment was not able to prevent or even stabilize the disease (Sevin et al., 2018).

The same approach is under clinical investigation using a LV. Ten patients with a confirmed diagnosis of MLD and brain lesions seen on MRI were selected to receive intra-parenchymatous injections of the TYF-ARSA LV. The objectives were to assess safety of intracerebral injection (at 1 year) and disease progression (at 3 years) and results are pending (the study was due to be completed in late 2020) (NCT03725670).

### Intracerebral Gene Therapy for MPS: The Sanfilippo Experience

Sanfilippo syndrome, or mucopolysaccharidosis type III (MPSIII), is a lysosomal storage disease with predominant neurological manifestations and coarse features, caused by a deficiency in one of the four enzymes involved in the lysosomal degradation of heparan sulfate. MPSIIIA (mutations in the N-sulfoglucosamine sulfohydrolase (*SGSH*) gene) and MPSIIIB (mutations in the N-acetyl-alpha-glucosaminidase (*NAGLU*) gene) are the most common types, characterized by a large phenotypic heterogeneity (Héron et al., 2011; Delgadillo et al., 2013). Using the same kind of approach and nearly the same protocol as for MLD and Batten disease, we have evaluated the safety and efficacy of intracerebral GT in children with MPSIIIA and MPSIIIB. Four MPSIIIA children (aged between 32 and79 months) received an AAV 10 vector-carrying human *SGSH* and *SUMF1* (sulfatase modifying factor 1) cDNAs through bilateral injections into 12 sites in the white matter. Immunosuppressive treatment was added during the follow up. Safety of procedure was demonstrated. In the first results published, MRI showed atrophy which seemed to be stable in two patients and showed a tendency to increase over follow-up in two others. In all four children, the disease progressed through its natural history, although neuropsychological assessments during the initial follow-up suggested some improvement in behavior, attention, and sleep in all patients, particularly in the youngest patient (Tardieu et al., 2014).

In the MPSIIIB phase I/II clinical trial, four children aged 20–53 months were treated with 16 intraparenchymal deliveries of the rAAV2/5-hNAGLU vector, combined with immunosuppression. Compared to the natural history of MPSIIIA/B, a neurocognitive benefit was observed at 30 months of follow up, which was more marked in the youngest patient, suggesting age is an important factor for treatment efficacy. NAGLU catalytic activity was detected in the CSF at 30 months (15–20% of the levels observed in unaffected children), consistent with the sustained production of the therapeutic enzyme in the brain tissues. A subpopulation of circulating blood lymphocytes proliferating and producing TNFa in response to ex vivo exposure to NAGLU antigens emerged but declined at 30 months, suggesting the development of tolerance (Tardieu et al., 2017a).

### Other Routes of Administration for *in vivo* Gene Therapy (Intra-Cerebrospinal fluid, Intravenous): New Perspectives?

After intraparenchymal administration, the vector spread is relatively poor (3–6 mm in the mouse brain) (Cearley and Wolfe, 2006) which, at the scale of a human brain, ultimately leads to limited diffusion of the therapeutic protein, even if we take into account the possibility of cross-correction and axonal transport of the vector or protein. Additionally, this method requires multiple injections, with the risk associated with the neurosurgical procedure. An alternative and less invasive procedure that could enable widespread vector distribution in the CNS is to infuse AAV vectors into the CSF through intracerebroventricular, intra-cisterna magna, or lumbar intrathecal injections (Piguet et al., 2017; Deverman et al., 2018; Hinderer et al., 2018; Bey et al., 2020). Alternatively, intravascular injection is also a promising option, but requires the vector to cross the BBB and is likely more immunogenic. For intra-CSF or intravenous administration, AAV9 and AAVrh10 are currently the best serotypes and are already being used in clinical practice (Piguet et al., 2017; Deverman et al., 2018). Among new serotypes under preclinical evaluation, AAV-PHP.eB also seems very promising for the delivery of genes across the BBB after intravenous administration (Chan et al., 2017). Most clinical trials using IV or intra-CSF administration are currently under evaluation, with pending results.

Intracisternal/intrathecal GT is evaluated in MPSI, MPSII, and CLN6. A phase I/II open-label dose-escalation study (NCT03580083) has started in MPSI patients with the severe Hurler phenotype, characterized by facial coarsening, skeletal deformities, failure to thrive, visceromegaly, hearing loss, profound intellectual disability, cardiorespiratory failure and early death within the first 10 years of life (Parini et al., 2017). Hurler patients will receive 1x10^10^ or 5x10^10^ vg/g brain mass of RGX-111 (AAV9 capsid containing the α-l-iduronidase gene) intracisternally and be followed up for 2 years to assess the safety and efficacy. The same approach is being evaluated in MPS II, an X-linked recessive LSD caused by mutations in the iduronate-2-sulfatase (*IDS*) gene in which patients present in the first years of life with peripheral symptoms manageable with intravenous ERT, but two-thirds also display a progressive and severe CNS involvement (Muenzer et al., 2017). Specifically, a phase I/II multicenter open-label dose-escalation clinical trial has been designed to assess the safety and efficacy of RGX-121 (AAV9-IDS) in 12 pediatric subjects with severe MPS II. The children are to be assessed (safety and efficacy) for 2 years following treatment (NCT03566043). Further, in patients with mild-to-moderate variant of LINCL with mutations in the *CLN6* gene, AAV9-CLN6 is being administered intrathecally in 13 patients (aged 1 year or older) in a phase I–II clinical trial (NCT02725580), with a 2-year follow up (to assess safety and efficacy). Results from these two trials should be available by the end of 2021.

Intravenous GT trials are in progress in MPSIIIA, MPSIIIB, and GM1 gangliosidosis. In MPSIIIA (mutations in the N-sulfoglucosamine sulfohydrolase (*SGSH*) gene), after encouraging preclinical results obtained in rodents, an open-label, single-dose clinical trial was launched (NCT04088734). All participants will receive a single intravenous dose of ABO-102 scAAV9.U1a.hSGSH (3 X 10^13^ vg/kg). The target population includes MPS IIIA children with a DQ < 60 (middle/advanced stages of the disease). Primary endpoints will include safety parameters, CSF heparan sulfate levels and liver/spleen volumes. Secondary endpoints will analyze, among others, neurological parameters (neuropsychological evaluation, quality of life, and brain MRI findings). The estimated study completion date is December 2022. The same approach is being used in a clinical trial for MPSIIIB (NCT03315182), supported by positive preclinical results. This study is enrolling patients from birth to 2 years or children > 2 years with a minimum DQ of 60 or above. This is a dose-escalation study with three groups (low-, medium- , and high-dose groups receiving 2 X 10^13^, 5 X 10^13^, and 10^14^ vg/kg, respectively). Primary endpoints include safety parameters and change from baseline in the age equivalent developmental score (compared with Natural History Study data), 2 years after treatment. Final results are expected by late 2022. Lastly, in a phase 1/2 safety and efficacy clinical trial (NCT03952637), AAV9/GLB1 will be administered by intravenous infusion to subjects with type II GM1 gangliosidosis (ages 2–12 years) or patients aged 6 months to 1 year with a type I GM1 gangliosidosis. The primary objective is to assess the safety of the procedure and secondary objectives include assessment of motor function, developmental change on the Vineland Adaptive Behavior Scales, modification in brain volume using a 3T MRI, and CNS metabolite levels by magnetic resonance spectroscopy. Results are expected in 2024.

#### *Ex vivo* Gene Therapy for Lysosomal Storage Disorders With Neurologic Involvement

As mentioned earlier, the long-term benefits of allogeneic HSCT in CNS disease are mediated by the replacement of brain microglial cells derived from donor bone marrow myelomonocytic cells. In the brain, donor-derived cells become an effective source of functional enzyme and cross-correction resident brain cells but also allow the restoration of normal microglia activity that may act on neuroinflammation. In *ex vivo* GT, patient HSCs are transduced *ex vivo* by a vector carrying the therapeutic gene and transplanted back into the patient following procedures such as those used in hematopoietic stem-cell transplantation (although in this case, the transplant is made up of autologous genetically modified cells). This leads to the progressive replacement of deficient microglia with microglia overexpressing the transgenic therapeutic proteins after hematopoietic reconstitution. For HSC-GT, the vectors of choice are LVs, derived from the human immunodeficiency virus type 1 (HIV-1), that have the ability to infect nondividing cells and demonstrated a low risk of insertional mutagenesis (Biffi, 2017; Piguet et al., 2017; Cavazzana et al., 2019; Lamsfus-Calle et al., 2020).

The best candidates for HSCT or HSC-GT (hematopoietic stem-cell gene therapy) are disorders in which microglia function is primarily affected, such as X-linked adrenoleukodystrophy (X-ALD). In accordance with this, HSCT has been a life changer in X-ALD for over 25 years and can arrest the neuroinflammatory demyelinating brain process, provided the procedure is performed at an early stage of the disease (Kemp et al., 2012; Raymond et al., 2019). *Ex vivo* GT using an LV was developed with encouraging preclinical results and launched the clinical practice in 2006 (Cartier et al., 2009). A clinical trial is ongoing is in children with cerebral ALD, who are candidates for allogeneic HSCT. Early results suggest that HSCT-GT may be a safe and effective alternative to allogeneic HSCT in boys with early-stage cerebral ALD (Eichler et al., 2017).

After ALD, MLD is the second inherited leukodystrophy for which HSC-GT has been developed. After promising results obtained in MLD mouse models (Biffi et al., 2004; Biffi et al., 2006), a phase I/II clinical trial of HSC-GT was initiated in 2011, results providing strong evidence of clinical benefit in presymptomatic patients with late infantile MLD (LI-MLD) and pre-or early-symptomatic patients with early-juvenile MLD (EJ-MLD) (Biffi et al., 2013; Sessa et al., 2016). HSC-GT–treated patients showed good hematopoietic reconstitution, stable engraftment of transduced HSC cells, and sustained ARSA activity in the blood and CSF. In all patients except one, the treatment prevented disease onset or halted disease progression, with an increase and/or stabilization in motor and cognitive performance and improved MRI findings, compared to those in untreated children. Another important finding was the effect on peripheral neuropathy, which is known to be poorly prevented by allogeneic HSCT. Available results for 33 patients (18 LI; 15 EJ) were presented at the WORLDSymposium in 2020, with a follow-up from 1 month to 7.5 years. All patients showed stable engraftment of gene-corrected cells, sustained restoration of ARSA activity in the hematopoietic system and cerebrospinal fluid. The majority of presymptomatic patients displayed long-term stabilization of motor function, many within normal range and most treated patients showed normal cognitive development (Fumagalli et al., 2020). These very promising results let us consider that HSCT-GT may become the standard of care for patients with an early form of MLD at a presymptomatic stage (Penati et al., 2017). A clinical trial has just started to evaluate the effect of this treatment in patients with late juvenile forms of MLD at pre- or early-symptomatic stages (NCT04283227).

Based on the same approach and considering the limitations of allogeneic transplantation in MPSI, HSCT-GT has been evaluated in the mouse model of the disease, providing evidence of a potential benefit (Visigalli et al., 2016). Further, a phase I/II clinical trial has started in Hurler disease. This trial will include eight patients, aged 1 month to 11 years, with a Lansky index of at least 80% (i.e., restricted in strenuous play, otherwise active). Patients will be followed up to 2 years for assessment of safety and efficacy parameters (NCT03488394).

### Central Nervous System Gene Therapy: Immune Response Against the Capsid and/or the Transgene

In *ex vivo* gene therapy, patients are exposed to immune suppressive conditioning regimens in order to allow engraftment of transduced HSPCs, thus the likelihood of an immune responses at the time of transplant is low (Annoni et al., 2019). On the opposite, neuroinflammation due to an immune response against the vector may occur after *in vivo* GT, both when the vector is injected systemically (into the blood) and when it is injected directly into the CNS (intra-CSF or intraparenchymal administration). The immune response may be triggered against the vector (preexisting and/or recall responses to the wild-type virus from which the vector is engineered) or against the transgene product itself and may interfere with the treatment efficacy. It may result from immune system activation involving monocytes/macrophages, and B and/or T cells and secretion of immune-modulatory products such as chemokines and cytokines, which might damage the BBB allowing the entry of innate and adaptative immune cells. Inflammatory mediators such as chemokines/cytokines could also stimulate resident brain cells such as astrocytes and microglia which would indirectly exacerbate neuroinflammation while peripheral immune cells that have entered would produce direct neurotoxicity. Direct administration of a vector into the CNS could also directly stimulate resident brain cells and similarly result in direct toxicity towards neurons or indirect neuroinflammation following the secretion of cytokines/chemokines, which might open the BBB leading to invasion and activation of systemic immune cells. Additionally, the local environment could have an impact, that is, when neuroinflammation is already present in the brain, as is the case of neuropathic LSDs, particularly at advanced stages of CNS disease (Rigante et al., 2017; Fiorenza et al., 2018).

Wild-type AAV infections are widely distributed in the human population, with initial exposure often occurring early in childhood, leading to humoral and cellular immune responses directed against the AAV capsid (Vandamme et al., 2017). After *in vivo* GT, the immune response against the viral capsid has been extensively studied in rodents and large animal models, as well as in clinical trials using peripheral administration of therapeutic vectors (Nayak and Herzog, 2010; Mingozzi and High, 2013). People who have been previously exposed to AAV vectors developed specific humoral and cellular immune response against the AAV capsid (Calcedo et al., 2011; Vandamme et al., 2017), and in patients previously infected with the same serotype of AAV, presence of antibodies before GT may block viral transduction (Peden et al., 2004; Wang et al., 2018), thereby limiting the production of the transgene and reducing treatment efficacy. This can be ruled out by testing for the presence of antibodies against the serotype used for gene transfer and by selecting patients with negative or weakly positive serology and/or administering immunosuppressive treatment after GT.

An immune response against the transgene itself is also a major concern (Mingozzi and High, 2013; Martino and Markusic, 2020). The immune system of children with a null mutation, who do not produce the normally secreted protein, will incorrectly identify the missing endogenous protein produced by the vector as a foreign body and develop a humoral and cellular immune reaction against it. These patients are classified as cross-reactive immunological material negative (CRIM-) and immunosuppression is frequently warranted in these cases as used in Pompe disease (Doerfler et al., 2016). Patients expressing a mutated enzyme with residual activity often react against the therapeutic recombinant enzyme following ERT, as observed in MPSI; however, after 2 years of treatment, patients seemed to developed immune tolerance to alpha-l-iduronidase (Kakavanos et al., 2003).

Major advantages of direct injection of the vector into the CNS are the direct access to the tissue where the transgene is most needed and the requirement for a lower amount of vector to achieve a clinical effect, which could indirectly minimize the immune response. Nevertheless, this would not prevent the appearance of systemic immune cell activation producing antibodies against the vector as observed in NHP studies using AAV9 or AAVrh10 delivered to the cisterna magna (Hordeaux et al., 2018; Bey et al., 2020) and in MPS large animal models (Ellinwood et al., 2011). These studies showed that the use of an immunosuppressive regimen allowed the expression of the vector and its delivery throughout the brain and disease correction in treated animals.

These preclinical results led to the addition of an immunosuppressive regimen during and after intracerebral vector administration in clinical trials for MPSIIIB and MPSIIIA (Tardieu et al., 2014; Tardieu et al., 2017b). In the MPSIIIB trial, the AAV5-NAGLU vector was detected from the end of its administration in the blood up to 48 h, suggesting the possibility of peripheral immune cell activation. Nevertheless, neutralizing antibodies (Nabs) against AAV5 were not observed at baseline or during follow up. Baseline immunophenotyping did not show any specific cellular activation but proliferation tests with the recombinant transgene (recombinant NAGLU) against systemic immune cells (TCD4/CD8) induced a detectable proliferative response of CD4 and CD8 cells when compared to baseline, suggesting activation of immune cells against the missing transgene despite immunosuppressive therapy. No inflammatory lesions were observed on MRI in any of the four treated children. These results seem to support the view that immunosuppression is needed in these models of GT.

In the MLD trial using intracerebral administration of the AAVRH10-ARSA vector, patients did not receive immunosuppressive drugs, except for corticosteroids given from Day-1 to Day+10 after administration of the therapeutic vector (Sevin et al., 2018). The AAVrh.10-hARSA genome was detected in urine up to Day+2 and in blood up to Month+3 after treatment. Levels of anti-AAV neutralizing antibodies rose up to Month+12, then tended to stabilize or decrease. There was no evidence of any humoral or cellular immune response against the ARSA transgene and no biochemical evidence of CSF inflammation (no increase in white blood cell counts or significant modification in cytokine patterns). Nonetheless, in some patients, hyperintense T2-weighted signals restricted to the injection sites were detected from Month+3 and remained stable thereafter, though there was no evidence of a clinical impact. We observed a significant increase in the ARSA activity in the CSF of treated patients that remained stable with time, suggesting that there was no decrease in the recombinant ARSA activity in the CSF due to an immune response against the recombinant ARSA. Nonetheless, because results were somewhat disappointing (lack of efficacy), we are unable to rule out that, around the injection site where the vector is concentrated and ARSA expression is likely the highest, some degree of immune reaction may have occurred that decreased the efficacy of the treatment. In the view of the results of these two trials (MPSIIIB and MLD), even though they do not allow us to draw formal conclusions, it seems reasonable to consider immunosuppressive therapy for *in vivo* GT, whatever the route of administration. Alternatively, as in the clinical trial for MPSIIIA and MPSIIIB, using intravenous administration of an AAV9 vector, exclusion should be discussed for patients with evidence of a humoral/cellular response against the AAV9 vector at baseline, as well as patients with two nonsense or null variants on genetic testing of the NAGLU gene.

### Lessons and Perspectives

In this review, we have attempted to clarify the rationale for using the “gene therapy” tool in neuropathic LSDs, describe the different approaches currently available and outline the clinical trials underway. We recognize that this review is likely incomplete and certainly is not able to cover all aspects of GT in LSDs. Due to the large amount of preclinical data from rodents and large animals, which are often very encouraging (negative results being less likely to be published), we have deliberately focused on clinical trials. Though currently results have only been reported for a small number of these trials, focusing on clinical trials may already help us to address questions raised in rodents and large animals that often do not exactly mimic the human disease.

The first conclusion is that neuropathic LSDs are particularly attractive candidates for GT. They are monogenic diseases and for most of them, there is a window of opportunity for therapeutic intervention in patients before onset of neurologic symptoms, allowing early intervention at a pre- or early-symptomatic stage. Correction of a small subset of neural cells should be amplified by cross-correction of adjacent cells and we may expect that the amount of enzyme required to achieve therapeutic correction may be around 10–15% of the normal range. Even if the GT protocol is primarily designed to target the CNS, peripheral involvement (if any) may be amenable to combined treatment with intravenous ERT, but also targeted by gene transfer beyond the CNS [after peripheral administration through HSCT-GT or intravenous GT, but also after intra-CSF administration if the vector passes into the bloodstream (Bey et al., 2020)]. Taken together, these findings lead to the expectation of a beneficial effect for patients.

What is the best route of administration? It is currently difficult to answer this question, most of the trials still being underway. *In vivo* and *ex vivo* GT have pros and cons that should be considered on a case-by-case basis for each disease. Both require only a one-shot treatment, with no need for readministration. *In vivo* GT just replaces the deficient gene, while *ex vivo* GT additionally resets the immune system and provides an *in situ* supply of donor-derived cells which may modify the environment and modulate immunity in the CNS. The effects of *in vivo* GT are rapid (2–3 weeks), whereas the impact of HSCT-GT is only noted some 12 months after treatment, due to the slow turnover of microglia. In terms of safety, the main concern regarding the use of *in vivo* GT is the possibility of an immune response against the vector or transgene, whereas with HSCT-GT, in addition to the fatigue and risks associated with myeloablative conditioning, patients should be monitored carefully for the development of insertional mutagenesis.

For intraparenchymal GT, based on results available in children, we can conclude that 1) the procedure is well tolerated, without serious adverse effects related to the surgery or to the GT vector itself; 2) the efficacy of the procedure and functionality of the vector (i.e., its capacity to transduce cells in the CNS, that are able to secrete a functional enzyme detected in the CSF) are attested to by significant expression of the recombinant enzyme in the CSF having been observed (20–70% in the MLD trial and around 15% in the MPSIIIB trial); and 3) the clinical efficacy is variable (lack of efficacy in MLD and a trend toward positive results in the LINCL, MPSIIIB, and MPSIIIA trials, for which long-term evaluation is currently underway). Several drawbacks may explain these mitigated results, including but not limited to the 1) use of an insufficient dose of vector; 2) failure to target the right cells, at the right place and at the right time; 3) lack of sufficient cross-correction of non-transduced cells in the human brain (cell type or amount of cells); and/or 4) misjudgment of the immune response against the therapeutic transgene. In addition, the design of the trials and particularly the inclusion criteria (the first trials allowed inclusion of patients with relatively advanced disease) may have a significant impact on the final results. Compared to the relative invasiveness of intracerebral GT, the most recent trials for *in vivo* GT involve less invasive routes of administration (intravenous, intra-CSF). For these trials, results have yet to be published. Intravenous administration may be by far the easiest route for administering *in vivo* CNS GT, facilitated by the emergence of new serotypes of AAV able to cross the BBB with a high tropism for the CNS (Chan et al., 2017). Nonetheless, it may be associated with complex issues (in particular, immune response and off-target expression) and requires a large amount of vector to be administered (Piguet et al., 2017).

Very encouraging results have been obtained using *ex vivo* GT in early-onset forms of MLD (Sessa et al., 2016; Penati et al., 2017). If performed at a presymptomatic stage in late infantile patients or in pre/early-symptomatic patients with early juvenile MLD, it may prevent or stabilize the disease. These results are markedly better than those obtained with allogeneic HSCT in patients with early-onset forms of MLD, even if presymptomatic. One hypothesis to explain this difference is that very high levels of ARSA enzyme might be required for the correction of the lysosomal defect in the CNS, HSCT-GT providing supraphysiological levels of recombinant enzyme, compared to the physiological levels obtained with allogeneic HSCT. This is an important result in the field of MLD, for which no treatment is currently available, particularly for these early-onset forms of the disease. On the other hand, HSCT-GT has no positive effect once patients are symptomatic in the case of rapidly progressing late infantile MLD, and for patients, there is an urgent need for alternative treatment options. HSCT-GT is currently under evaluation in patients with late-juvenile MLD, who are presymptomatic or very early symptomatic at the time of the treatment. It will be interesting to see whether, in these patients, HSCT-GT outperforms allogeneic HSCT, which is insufficient to prevent all the symptoms, in particular, peripheral nerve and cerebellar involvements, but does clearly modify the natural history of the disease.

Depending on the disease course, a cascade of secondary events may occur in LSDs, in addition to the storage of undegraded material, and these may hamper the efficacy of the treatment. These include increased oxidative stress, perturbed calcium homeostasis, microglia activation, and astrogliosis. Intuitively, all these perturbations may impact the efficacy of GT to some extent. Chronic neuroinflammation associated with microglial and astrocyte activation is a hallmark of neuropathic LSDs and may play a role in neuropathogenesis, although the timing and intensity of inflammatory events likely differ in each LSD (Bosch and Kielian, 2015). Administering a therapeutic gene in a hostile pro-inflammatory environment may lead to a decrease in transduction or impair survival of corrected cells and have a negative impact in the case of *in vivo* GT, particularly if treatment is performed at a late stage of the disease. And finally, one may question whether replacing a gene/enzyme in a hostile environment and a diseased brain would be sufficient to have an impact on the disease, or whether additional combined treatments should be considered.

Interestingly, in MLD, Krabbe, or MPSI, allogeneic HSCT may have some clinical benefit, even if insufficient to halt the disease. As well as providing a source of functional enzyme (as *in vivo* GT), donor-derived cells that migrate into the brain after HSCT or HSCT-GT may have an additional effect on neuroinflammation and/or secondary mechanisms involved in neurodegeneration. This second mechanism of action could be helpful, if not mandatory, at least for LSDs in which neuroinflammation is evident, arguing in favor of HSCT-GT over *in vivo* GT. HSCT-GT will additionally allow overexpression of the recombinant enzyme unlike conventional allogeneic HSCT. An alternative would be to combine allogenic HSCT and *in vivo*-GT, making it possible to simultaneously consider a rapid supply of the deficient enzyme (*via* GT) and an action on neurodegeneration (*via* HSCT). At the time of submission of this article, a clinical trial has just started combining intravenous AAVRH10-GT and HSCT in presymptomatic patients affected with the infantile form of Krabbe disease (NCT04693598).

Taken together, the first results obtained in clinical trials using GT in neuropathic LSDs give real hope that this strategy will help patients, particularly if they are treated at a presymptomatic or early-symptomatic stage of their disease. This underlines the need for early diagnosis, including familial screening of index cases, but also the implementation of newborn screening for diseases in which effective therapies are likely to become available relatively soon, such as MLD and some MPS.

Nonetheless, improvements will likely be required, such as engineering capsids and promoters, to enhance CNS transduction and cell-type selectivity, avoiding off-target expression (i.e., using miRNA target sequences), optimizing the dosing and route of administration, and modifying capsid surface epitopes through capsid engineering to evade and manage immune response [for review, see (Chan et al., 2017; Piguet et al., 2017; Deverman et al., 2018; Perez et al., 2020)]. Additionally, the emergence of new gene therapy tools, particularly genome editing based on the CRISPR/Cas9 system, will likely be a promising alternative for the treatment of disorders associated with genome mutations, in particular LSDs. And finally, at the time of clinical trial design, it will be crucial to select the appropriate patient population (sample size and control group), the right dose of vector, the appropriate endpoints for safety and efficacy, the magnitude of the treatment effect and a suitable length of follow-up to be able to observe this effect, and, ultimately, assess the overall benefit-to-risk ratio.
